# Value of Contrast-Enhanced Ultrasound in Mummified Thyroid Nodules

**DOI:** 10.3389/fendo.2022.850698

**Published:** 2022-03-18

**Authors:** Sijie Chen, Kui Tang, Yi Gong, Fei Ye, Liyan Liao, Xiaodu Li, Qi Zhang, Yan Xu, Rongsen Zhang, Chengcheng Niu

**Affiliations:** ^1^ Department of Ultrasound Diagnosis, The Second Xiangya Hospital, Central South University, Changsha, China; ^2^ Research Center of Ultrasonography, The Second Xiangya Hospital, Central South University, Changsha, China; ^3^ Department of Thyroid Surgery, The Second Xiangya Hospital, Central South University, Changsha, China; ^4^ Department of Pathology, The Second Xiangya Hospital, Central South University, Changsha, China

**Keywords:** contrast-enhanced ultrasound (CEUS), mummified thyroid nodules (MTNs), papillary thyroid carcinomas (PTCs), thyroid ultrasonography, fine-needle aspiration (FNA)

## Abstract

Mummified thyroid nodules (MTNs) are rarely reported and are usually misdiagnosed as malignant nodules. This article first reviewed the contrast-enhanced ultrasound (CEUS) enhancement features of 218 MTNs and classified them into three (A, B, C) patterns. The A pattern MTNs show linear hypo-enhancement, the B pattern MTNs show heterogeneous hypo-enhancement, and the C pattern MTNs show no enhancement in thyroid nodules. The A and C pattern enhancements of MTNs demonstrated a high specificity compared with the enhancement of previously reported typical papillary thyroid carcinomas (PTCs). To further study the B pattern MTNs, 24 B pattern MTNs and 42 PTCs were enrolled in this study, and CEUS parameters for each nodule were evaluated. Univariate analysis indicated that compared with PTCs, the B pattern MTNs more frequently exhibited heterogeneous hypo-enhancement and clear margins after clearance (p <0.05). A multivariate analysis revealed that heterogeneous hypo-enhancement and clear margins after clearance were independent characteristics related to the B pattern MTNs for differentiating them from PTCs (p <0.05). Thus, preoperative CEUS may provide more important information for distinguishing MTNs from malignant thyroid nodules to avoid surgical excisions or unnecessary fine-needle aspiration (FNA).

## Introduction

High-frequency ultrasound (US) is an important imaging modality for detecting thyroid nodules and differentiating malignant thyroid nodules from benign thyroid nodules (1–4). However, some previously proven benign thyroid nodules may spontaneously shrink in size and show morphologic changes of suspicious malignancy on US examinations spontaneously over time or result from thyroid nodule fine-needle aspiration biopsy (FNAB), percutaneous ethanol injection, or radiofrequency ablation (5–8). These changes include strong hypoechogenicity, solid components, irregular margins and microcalcifications. These features are collectively referred to as the thyroid nodule mummification process, and the thyroid nodules are called mummified thyroid nodules (MTNs) (5, 9).

According to the Thyroid Imaging Reporting and Data System (TI-RADS), five US suspicious features were applied to categorize the thyroid nodules, whereas the MTNs usually have more than two suspicious features and are classified as TIRADS scores 4b to 5 (9). Thus, some patients with MTNs undergo fine-needle aspiration (FNA) or even surgery, which results in the potential for over-management (10).

Contrast-enhanced ultrasound (CEUS) could provide improved characterization of dynamic microvessel perfusion in the differential diagnosis of focal thyroid nodules compared with color Doppler US, which could help to differentiate malignant thyroid nodules from benign thyroid nodules (9, 11–13). However, MTNs with the same conventional US appearance may have different CEUS features according to our current study. In this study, we first reviewed the CEUS enhancement features of 218 MTNs and classified them into three (A, B, C) patterns. A pattern with linear hypo-enhancement and C pattern with no enhancement could easily diagnose MTNs combined with a previous clinical history (14). However, the B pattern with heterogeneous hypo-enhancement was very similar to the enhancement of typical papillary thyroid carcinomas (PTCs), which is necessary to identify PTCs from malignant thyroid nodules, especially in the absence of clinical history. The purpose of our study was to highlight the different CEUS features of MTNs that should help to differentiate benign collapsed thyroid nodules from malignant thyroid nodules.

## Materials and Methods

### Patients

The study was approved by the Ethical Committee of the Second Xiangya Hospital of Central South University in China and was performed in accordance with the Declaration of Helsinki for human studies. The requirement of informed consent from human subjects is occasionally waived by IRBs for protocols that include a retrospective review of images acquired for clinical diagnostic purposes. From November 2016 to November 2021, 469 patients with 469 suspicious MTNs who received conventional US and CEUS examinations were retrospectively enrolled in this case-control study. The inclusion criteria were as follows: (1) patients with thyroid nodules were suspected to be malignant according to conventional US; (2) nodules have a clinical history with no suspicious features; and (3) nodules were confirmed as benign by pathology after surgery or by FNAB. Among them, 251 patients with no follow-up US examinations or further FNAs were excluded. Finally, 218 patients with 218 MTNs were included in this study. Then, the MTNs were classified into 3 patterns according to the different CEUS enhancement types.

As a control group, 236 patients with 236 malignant thyroid nodules who received conventional US and CEUS examinations from July 2020 to January 2021 were recruited in this study with the inclusion criteria: (1) patients with thyroid nodules confirmed as PTCs by pathological examination after surgery; (2) suspicious nodules for malignancy in conventional US; (3) all nodule sizes in the control group were less than 15 mm, which is consistent with that noted in the study group; and (4) hypo-enhancement of nodules, which is consistent with that noted in the study group. Four patients with 4 thyroid nodules were excluded because they had different types of thyroid cancers: 3 follicular and 1 medullary thyroid cancers. A total of 127 patients with 127 thyroid nodules were excluded due to a nodule size greater than 15 mm, and 63 patients with 63 thyroid nodules were excluded due to hyper- or iso-enhancement. Finally, 42 patients with 42 PTC nodules were included in this study.

### Conventional US

A Siemens Acuson S3000 US scanner (Mountain View, CA, USA) was used for conventional US and CEUS. The equipped 9L4 and 18L6 linear array transducers were used for conventional US, and the 9L4 for CEUS. All selected thyroid nodules were evaluated by conventional US and classified according to the Kwak TI-RADS (4). Five US suspicious features (solid component, marked hypo- or hypo-echogenicity, irregular margins, taller-than-wide shape, and microcalcifications) were applied to categorize the thyroid nodules as TI-RADS category 3 (no suspicious US features), 4a (1 suspicious US feature), 4b (2 suspicious US features), 4c (3 or 4 suspicious US features), and 5 (5 suspicious US features).

### CEUS and Analysis

CEUS performed technology and the ultrasound contrast agents were the same as our previous studies (9, 11–13). The CEUS videos were acquired and time-intensity curves (TICs) of the thyroid within selected regions of interest (ROIs) were analyzed according to our previous study (11). We simply divided the CEUS time into two phases: the early enhancement phase (from the microbubbles entering the nodule until reaching the peak intensity) and the late enhancement phase (the microbubbles in the nodule begin to fade away until they disappear). After comparison with the surrounding thyroid parenchymal enhancement, the contrast enhancement features were classified as follows: enhancement type (no enhancement, linear enhancement, which means marked hypo-enhancement, homogeneous hypo-enhancement or heterogeneous hypo-enhancement), arrival time (the time of microbubbles arriving at the nodule compared with the thyroid parenchymal tissue), peak intensity (PI, expressed as a percentage), and area under the curve (AUC, expressed in percentage by seconds). The PI and AUC of the nodules are reported as indices by the ratio of the ROI of the nodules to the ROI of the thyroid parenchymal tissue according to our previous studies (11, 12).

### Reference Standard

FNA Bethesda cytology (BC) diagnoses were divided into six categories according to the Bethesda System (1). For PTCs, the histopathological results after surgery were used as the reference standard. For MTNs, the FNA results classified as BC 2 within at least 6 months of the follow-up US were used as the reference standard in addition to the histopathologic results after surgery.

### Statistical Analysis

SPSS version 21.0 software (SPSS, Chicago, IL, USA) was used for statistical analysis. Continuous data are presented as mean and standard deviation and were compared by the independent t-test. Categorical data are presented as percentages and were analyzed by the Chi-square test. Binary logistic regression was used to assess significant CEUS features and their independent association with MTNs. P <0.05 means the difference has statistical significance.

## Results

A total of 218 patients with MTNs were included in the analysis. Twelve (5.5%) patients underwent total thyroidectomy for PTC on the other lobe, and the MTNs were demonstrated to be nodular goiter by paraffin section examination. Fifteen (6.9%) patients who underwent partial thyroidectomy for cytopathologic examination disclosed atypia of unknown significance by ultrasound guided FNA and requests for surgery from patients, and the nodules were benign, as confirmed by histopathology. The remaining 191 (87.6%) patients underwent FNA. In total, 176 lesions were proven to be benign and 15 were considered inadequate specimens at the initial FNA and underwent repeat FNA. All nodules were consistent with benign thyroid nodule collapse changes (**Table 1**).

**Table 1:** Diagnostic Performance of the MTNs by the Kwak TI-RADS.

TI-RADS score	Number of malignant US features	MTNs (n = 218)	Pathologic method	
			Surgery (n = 27)	FNA (n = 191)
4b	2	28	0	28
4c	3–4	142	22	120
5	5	48	5	43

According to the TI-RADS diagnostic classification by Kwak et al. (4), the US performances of the MTNs were classified (**Table 1**). In this study, all MTNs had at least 2 suspicious US features: a solid component and hypoechogenicity or marked hypoechogenicity. Thus, 28 (12.9%) patients were classified as TI-RADS category 4b, 142 (65.1%) patients as TI-RADS category 4c, and 48 (22.0%) patients as TI-RADS category 5.

**Figure 1:**
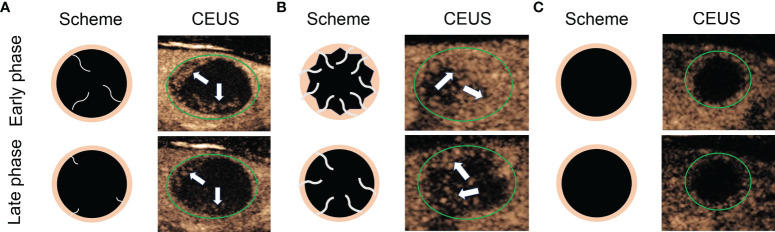
Diagram shows different CEUS enhancement patterns of mummified thyroid nodules. **(A)** Early phase of CEUS shows a linear perfusion of microbubbles with a clear margin compared with surrounding enhanced thyroid parenchymal tissue, and the late phase of CEUS shows the microbubbles faded away with a clear margin. **(B)** Early phase of CEUS shows a heterogeneous hypo-enhancement of microbubbles perfusion with a blurred margin compared with surrounding enhanced thyroid parenchymal tissue, and the late phase of CEUS shows the microbubbles faded away with a clear margin. **(C)** Both early phase and late phase of CEUS show no microbubbles enter the thyroid nodules, which has a clear margin compared with surrounding enhanced thyroid parenchymal tissue. The green circles indicate the margin of the thyroid nodules, the white arrows indicate the microbubbles perfusion in the thyroid nodules.

**Figure 2:**
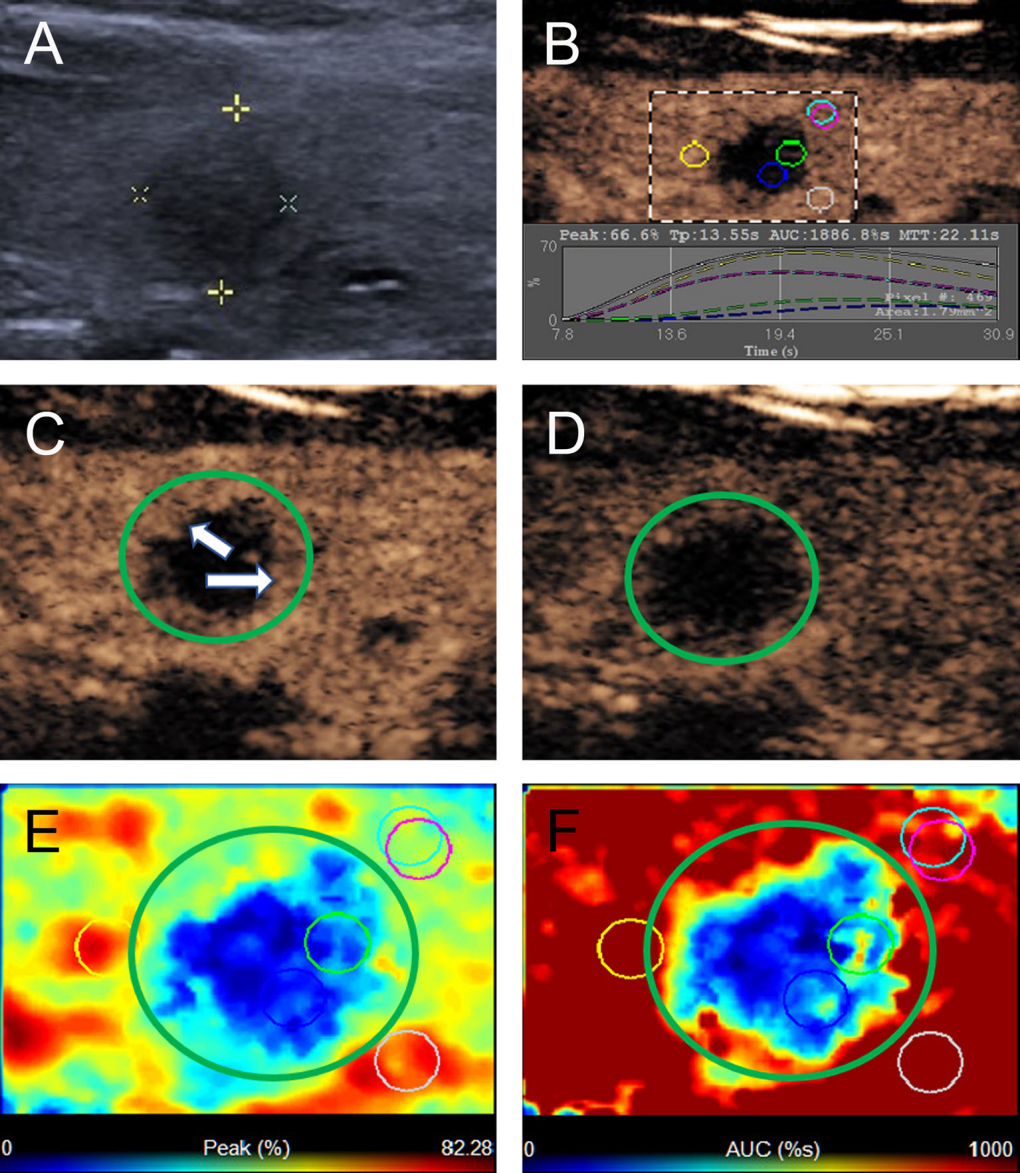
A 40-year-old woman with A pattern mummified thyroid nodule proven by FNAB. **(A)** Nodule showed solid component, hypoechogenicity, ill-defined margin and taller than wide shape (TI-RADS 4c). **(B)** Time-intensity curve showed the CEUS parameters of this nodule compared with peripheral thyroid parenchyma. **(C)** The early phase of CEUS showed a linear enhancement (white arrows) in the whole nodule (green circle). **(D)** The late phase of CEUS showed the microbubbles in the nodule fade away with a clear margin compared with peripheral thyroid parenchyma. **(E)** Parametric color map showed the values of peak for the nodule was totally deep blue compared with the surrounding yellow color of thyroid parenchyma, indicated the peak intensity of the nodule was markedly lower than peripheral thyroid parenchyma. **(F)** Parametric color map showed that the AUC for the nodule was totally deep blue with a little linear yellow on the peripheral of the nodule, indicated AUC of the nodule was obviously lower than peripheral thyroid parenchyma.

**Figure 3:**
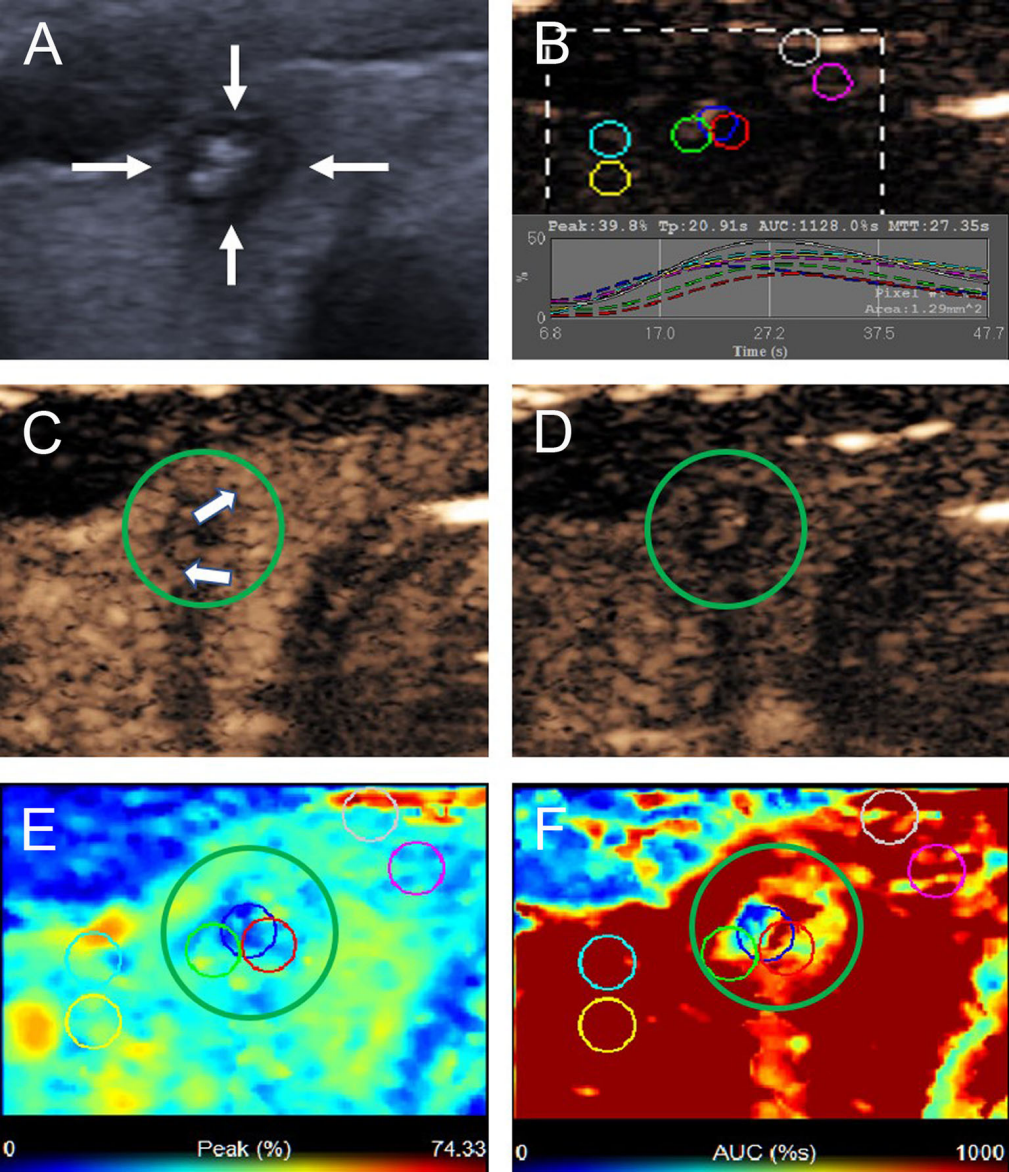
A 22-year-old woman with the B pattern mummified thyroid nodule proven by FNAB. **(A)** Nodule showed solid component, hypoechogenicity, ill-defined margin, taller than wide shape and microcalcification (TI-RADS 5). **(B)** Time-intensity curve showed the CEUS parameters of this nodule compared with peripheral thyroid parenchyma. **(C)** The early phase of CEUS showed a heterogeneous hypo-enhancement (white arrows) in the whole nodule (green circle). **(D)** The late phase of CEUS showed the microbubbles in the nodule fade away with a clear margin compared with peripheral thyroid parenchyma. **(E)** Parametric color map showed the values of peak for the nodule was uneven deep blue and blue, compared with the surrounding yellow and blue color of thyroid parenchyma, indicated the peak intensity of the nodule was lower than peripheral thyroid parenchyma. **(F)** Parametric color map showed that the AUC for the nodule was uneven deep blue and yellow color, compared with the surrounding red color of thyroid parenchyma, indicated AUC of the nodule was lower than peripheral thyroid parenchyma.

**Figure 4:**
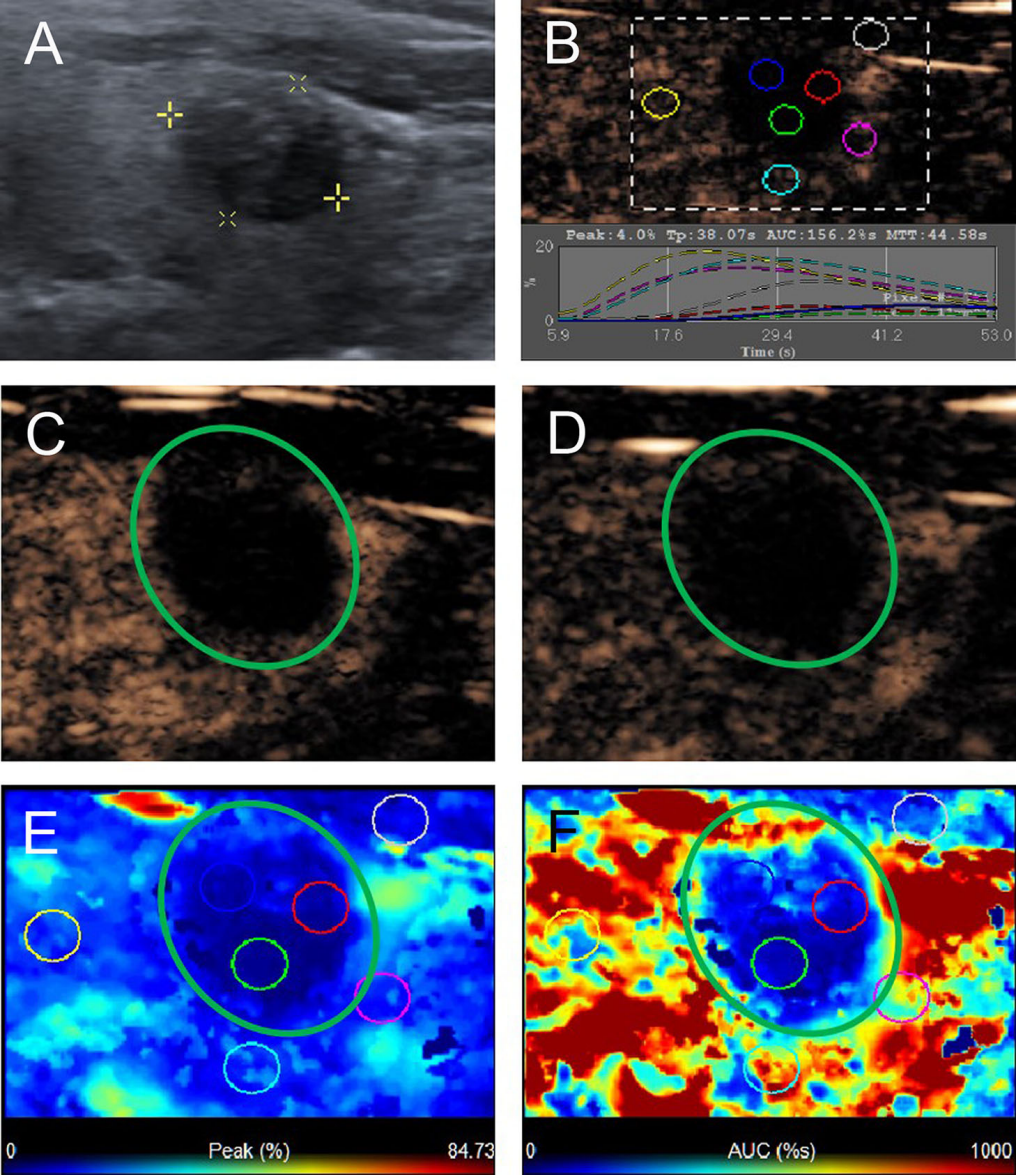
A 58-year-old man with C pattern mummified thyroid nodule proven by FNAB. **(A)** Nodule showed solid component and hypoechogenicity (TI-RADS 4b). **(B)** Time-intensity curve showed the CEUS parameters of this nodule compared with peripheral thyroid parenchyma. **(C)** The early phase and **(D)** the late phase of CEUS showed no microbubbles enter the nodule (green circle). **(E)** Parametric color map showed the values of peak for the nodule was completely deep blue, indicated the peak intensity of the nodule was markedly lower than peripheral thyroid parenchyma. **(F)** Parametric color map showed that the AUC for the nodule was totally deep blue compared with the surrounding red color of thyroid parenchyma, indicated AUC of the nodule was obviously lower than peripheral thyroid parenchyma.

According to the CEUS enhancement types, the MTNs were classified into 3 patterns (A, B, C patterns) (**Figure 1**). The A pattern enhancement of MTNs shows a linear perfusion of microbubbles (marked hypo-enhancement) with a clear margin compared with surrounding enhanced thyroid parenchymal tissue in the early enhancement phase, and the microbubbles faded away with a clear margin in the late enhancement phase (**Figure 2**). The B pattern enhancement of MTNs shows heterogeneous hypo-enhancement of microbubbles perfusion with a blurred margin compared with surrounding enhanced thyroid parenchymal tissue in the early enhancement phase, and the microbubbles faded away with a clear margin in the late enhancement phase (**Figure 3**). The C pattern enhancement of MTNs shows no microbubbles (no enhancement) entering the thyroid nodules, which has a clear margin compared with surrounding enhanced thyroid parenchymal tissue in both the early and late enhancement phases (**Figure 4**). The A and C pattern enhancements of MTNs demonstrated a high specificity compared with the enhancement of previously reported typical PTCs, and linear enhancement and no enhancement were almost never shown in the enhancement of PTCs. However, the B pattern enhancement of MTNs exhibited heterogeneous hypo-enhancement, which was also shown in most PTCs. Therefore, more CEUS features of the B pattern MTNs require further study to differentiate them from PTCs.

In this study, 149 patients with 149 A pattern MTNs, 24 patients with 24 B pattern MTNs and 45 patients with 45 C pattern MTNs were included in the analysis. To further study the B pattern MTNs, 42 patients with 42 PTCs were also included in the control group. The mean diameter of the B pattern MTNs was 8.46 ± 3.05 mm and ranged from 4 to 15 mm. The mean diameter of the PTCs was 7.62 ± 3.02 mm and ranged from 4 to 15 mm. The diameters of the nodules were not significantly different between the two groups (*p >*0.05, **Table 2**). The CEUS features of the B pattern MTNs and PTCs are summarized in **Table 2**. For both the B pattern MTN and PTC groups, all nodules showed hypo-enhancement, PI index <1 and AUC index <1, and no differences were noted between the two groups. Regarding the enhancement type, 23 (95.8%) nodules exhibited heterogeneous hypo-enhancement and only 1 (4.2%) nodule showed homogeneous hypo-enhancement in the B pattern MTNs, indicating that the microbubbles in most of the nodules were unevenly distributed. However, 29 (69.0%) nodules exhibited homogeneous hypo-enhancement and 13 (31.0%) nodules showed heterogeneous hypo-enhancement in the PTC groups, indicating that the microbubbles in most of the nodules were evenly distributed. A significant difference was noted between the two groups (*p <*0.05). Regarding arrival time, 23 (95.8%) nodules showed late arrival time in the B pattern MTNs, whereas 37 (88.1%) nodules showed late arrival time in the PTC group. These findings demonstrated that the microbubbles arriving at the nodules were later than those of the thyroid parenchymal tissue in most of the nodules in both groups, and no significant difference was noted between the groups (*p >*0.05). Regarding the margin after clearance, 20 (83.8%) nodules exhibited a clear margin after the microbubbles faded away, and only 4 (16.7%) nodules showed blurred margins after the microbubbles faded away in the B pattern MTNs. These finding indicates that the microbubbles vanished quickly in the whole nodule and left a clear margin with the thyroid parenchymal tissue. However, 39 (92.9%) nodules exhibited blurred margins after the microbubbles faded away and 3 (7.1%) nodules showed clear margins after the microbubbles faded away in the PTC groups, indicating that the microbubbles had a small dose retention in the nodules without an obvious disappearance in the late phase. A significant difference was noted between the two groups (*p <*0.05). Thus, the univariate analysis indicated that compared with the PTC nodules, the B pattern MTNs more frequently exhibited heterogeneous hypo-enhancement and clear margins after clearance (*p <*0.05).

**Table 2:** CEUS Characteristics of the B pattern MTNs and PTCs.

Characteristics	MTNs (n = 24)	PTCs (n = 42)	*p*
Size (mm)	8.46 ± 3.05	7.62 ± 3.02	0.683
Enhancement type			0.000*
Homogeneous hypo-enhancement	1 (4.2)	29 (69.0)	
Heterogeneous hypo-enhancement	23 (95.8)	13 (31.0)	
Arrival time			0.404
Early	1 (4.2)	5 (11.9)	
Late	23 (95.8)	37 (88.1)	
Margin after clearance			0.000*
Clear	20 (83.3)	3 (7.1)	
Blurred	4 (16.7)	39 (92.9)	

*p <0.05 was considered a significant difference.

A binary logistic regression analysis was performed for two statistically significant CEUS variables (p <0.05). The results indicated that heterogeneous hypo-enhancement (B = 2.655, odds ratio [OR] = 14.225, 95% confidence interval [CI] = 1.383–146.265, *p* = 0.026) and clear margin after clearance (B = 3.264, OR = 26.167, 95% CI = 4.771–143.512, *p* = 0.000) were independent characteristics related to the B pattern MTNs for their differentiation from PTC nodules (**Table 3**).

**Table 3:** Multivariate logistic regression analysis of CEUS characteristics related to the B pattern MTNs distinguishing from PTCs.

Characteristics	Partial regressioncoefficient, β	Odds ratio	95% Confidence interval	*P*-Value
Heterogeneous hypo-enhancement	2.655	14.225	1.383–146.265	0.026*
Clear margin after clearance	3.264	26.167	4.771–143.512	0.000*

*p < 0.05 was considered a significant difference.

## Discussion

Benign thyroid nodules may display morphologic changes with suspicious malignant US features over time, and the progression of benign thyroid nodule necrosis, desiccation, and subsequent collapse is called thyroid nodule mummification (5). Previous studies have shown that the typical MTNs have US imaging features, such as solid components, marked hypoechogenicity, punctate echogenic foci, double black-and-white peripheral halos and posterior shadowing (9). Comparison with previous images showing thyroid nodule shrinkage over time is useful for reaching the correct final diagnosis (5, 9, 15). Thus, some patients with MTNs could effectively avoid any unnecessary FNA or even surgical procedures, which maybe cause more severe complications (16–18).

In this study, according to the Kwak TI-RADS classification, at least 2 suspicious US features (solid component and hypo-echogenicity) were shown in all MTNs, so their classifications were at least a TI-RADS score of 4b. Of the 218 MTNs, 28 nodules had a TI-RADS score of 4b, 142 nodules had a TI-RADS score of 4c, and 48 nodules had a TI-RADS score of 5. Of 218 patients, 27 patients underwent total thyroidectomy or underwent total thyroidectomy, and 191 patients underwent FNA. However, if the patients have no clear clinical history, such as previous cystic or predominantly cystic thyroid nodules, this collapsed nodule with a high TI-RADS classification in grayscale US may have to undergo unnecessary FNA or even surgery.

CEUS exhibits significant value in the diagnosis of collapsing benign cystic or predominantly cystic thyroid nodules, especially combined with clinical history. No enhancement or scant punctate-linear enhancement in the whole or most areas of the nodules exhibits good specificity for MTNs (14). In this study, 218 patients with 218 MTNs were included and classified into 3 patterns (A, B, C) according to the different CEUS enhancement types. The A pattern CEUS of MTNs shows a linear perfusion of microbubbles with a clear margin compared with surrounding thyroid parenchymal tissue, which was consistent with the scant punctate-linear enhancement mode for the whole or most of the regions of the nodules (14). These nodules usually displayed fibrous tissue hyperplasia, cholesterol crystallization, foam tissue cells and lymphocyte infiltration in histopathology. The C pattern CEUS of MTNs shows no microbubbles entering the thyroid nodules, which have a clear margin compared with surrounding enhanced thyroid parenchymal tissue in both the early and late enhancement phases. This finding was consistent with no enhancement in previously reported studies (9, 14). The A and C pattern enhancements of MTNs demonstrated high specificity compared with the enhancement of previously reported typical PTCs, and linear enhancement and no enhancement were almost never noted in the enhancement of PTCs (14). However, the B pattern CEUS of MTNs shows heterogeneous hypo-enhancement of microbubbles perfusion with a blurred margin compared with surrounding enhanced thyroid parenchymal tissue in the early enhancement phase, and this hypo-enhancement mode is highly similar to the enhancement mode of PTCs (19, 20). In this study, heterogeneous hypo-enhancement occurred more frequently in the B pattern MTNs than in PTCs. In the late enhancement phase, the microbubbles faded away with a clear margin, which was an independent feature for MTNs compared with PTCs. This finding indicates that the microvessels of the MTNs were less abundant than those of PTCs and were typically distributed on the peripheral part of the nodule. This type of MTN will further collapse and lacks vasculature on the follow-up US examination over time in our current study. However, these findings have not been systematically reported to date. Therefore, familiarity with the CEUS features of these different enhancement modes suggests that MTNs may be helpful in reducing repeated FNA or unnecessary surgery.

This study had several limitations. First, most of the final diagnoses for MTN result used FNA with at least 6 months of follow-up US as the reference standard, which may lead to false negatives. Second, unavoidable selection bias may exist because the patients with suspicious US features of malignancy might not undergo surgery or further examination. Third, the control group of PTCs was chosen with a size less than 15 mm and hypo-enhancement for in line with the study group, which may cause unavoidable selection bias and inaccurate evaluation of the effect for CEUS features. Thus, a large-scale study with a multicenter prospective design is needed to clarify our data and provide the specificity and sensitivity of the reported CEUS findings.

## Conclusion

In conclusion, we first reviewed the CEUS enhancement features of 218 MTNs and classified them into three (A, B, C) patterns. The A pattern MTNs show linear hypo-enhancement with only scant microbubbles entering the thyroid nodule. The B pattern MTNs show heterogeneous hypo-enhancement and a small amount of microbubbles perfusion with uneven distribution in the thyroid nodule. The C pattern MTNs show no enhancement and have no microbubbles in the thyroid nodules. In addition, compared with PTCs, heterogeneous hypo-enhancement and clear margins after clearance were independent characteristics related to the B pattern MTNs for their differentiation from PTCs (p <0.05). Therefore, preoperative CEUS characteristics may provide more important information for distinguishing MTNs from malignant thyroid nodules to avoid surgical excisions or unnecessary FNAs.

## Data Availability Statement

The raw data supporting the conclusions of this article will be made available by the authors, without undue reservation.

## Ethics Statement

The studies involving human participants were reviewed and approved by the Ethical Committee of the Second Xiangya Hospital of Central South University in China. The patients/participants provided their written informed consent to participate in this study. Written informed consent was obtained from the individual(s) for the publication of any potentially identifiable images or data included in this article.

## Author Contributions

CN contributed to the conception and design of the work. SC and KT participated to data analysis and manuscript writing. YG, FY, LL, XL, QZ, YX, and RZ participated to data collection and follow-up of patients. All authors listed have made a substantial, direct, and intellectual contribution to the work and approved it for publication.

## Funding

This project was funded by the National Natural Science Foundation of China (81974267) and the Science and Technology Innovation Program of Hunan Province (2021RC3033).

## Conflict of Interest

The authors declare that the research was conducted in the absence of any commercial or financial relationships that could be construed as a potential conflict of interest.

## Publisher’s Note

All claims expressed in this article are solely those of the authors and do not necessarily represent those of their affiliated organizations, or those of the publisher, the editors and the reviewers. Any product that may be evaluated in this article, or claim that may be made by its manufacturer, is not guaranteed or endorsed by the publisher.
